# Integrated Analysis of Metabolomics Combined with Network Pharmacology and Molecular Docking Reveals the Effects of Processing on Metabolites of *Dendrobium officinale*

**DOI:** 10.3390/metabo13080886

**Published:** 2023-07-26

**Authors:** Lilan Xu, Si-Min Zuo, Mei Liu, Tao Wang, Zizheng Li, Yong-Huan Yun, Weimin Zhang

**Affiliations:** Key Laboratory of Tropical Fruits and Vegetables Quality and Safety for State Market Regulation, School of Food Science and Engineering, Hainan University, Haikou 570228, China; 22210832000036@hainanu.edu.cn (L.X.);

**Keywords:** *Dendrobium officinale*, processing, metabolites, network pharmacology, molecular docking

## Abstract

*Dendrobium officinale* (*D. officinale*) is a precious medicinal species of *Dendrobium* Orchidaceae, and the product obtained by hot processing is called “Fengdou”. At present, the research on the processing quality of *D. officinale* mainly focuses on the chemical composition indicators such as polysaccharides and flavonoids content. However, the changes in metabolites during *D. officinale* processing are still unclear. In this study, the process was divided into two stages and three important conditions including fresh stems, semiproducts and “Fengdou” products. To investigate the effect of processing on metabolites of *D. officinale* in different processing stages, an approach of combining metabolomics with network pharmacology and molecular docking was employed. Through UPLC-MS/MS analysis, a total of 628 metabolites were detected, and 109 of them were identified as differential metabolites (VIP ≥ 1, |log2 (FC)| ≥ 1). Next, the differential metabolites were analyzed using the network pharmacology method, resulting in the selection of 29 differential metabolites as they have a potential pharmacological activity. Combining seven diseases, 14 key metabolites and nine important targets were screened by constructing a metabolite–target–disease network. The results showed that seven metabolites with potential anticoagulant, hypoglycemic and tumor-inhibiting activities increased in relative abundance in the “Fengdou” product. Molecular docking results indicated that seven metabolites may act on five important targets. In general, processing can increase the content of some active metabolites of *D. officinale* and improve its medicinal quality to a certain extent.

## 1. Introduction

*Dendrobium officinale* (*D. officinale*) is a precious edible and medicinal plant of the *Dendrobium* Orchidaceae genus [1,2], and its product generated by hot processing is named “Fengdou”. “Fengdou” has been used for a long time in China and is known as “the first of the nine immortal herbs in China” [3]. Many studies have shown that *D. officinale* is rich in polysaccharides, flavonoids, alkaloids and so on [4,5]. A summary of reports has shown that *D. officinale* has many kinds of physiological activities, such as anticancer [6,7,8], hypoglycemic [9], anti-inflammatory [10], antihyperlipidemic, antihypertension, anticoagulant, tumor-inhibiting and immunity-improving activities [11]. Modern pharmacological research has demonstrated that metabolites are the material basis for *D. officinale* to exert physiological activities [12,13].

However, it was determined that the moisture content of fresh stems of *D. officinale* is as high as 96%, and the outer part of *D. officinale* is wrapped with a tough and dense cuticle, which can effectively prevent water from escaping. In addition, *D. officinale* is rich in polysaccharides; as a result, fresh stems can then maintain vigorous life activities after harvesting. If *D. officinale* cannot be processed in time, it will not only mildew but also easily sprout, which will result in the loss of nutrients and then lead to a serious decline in the quality of medicinal materials [3,14]. Therefore, processing is very important for *D. officinale*.

The processing method of *D. officinale* has been listed in the 2015 edition of the *Chinese Pharmacopoeia*, which is widely used in actual production. However, it is worth noting that the heating time and temperature are not clearly specified in this method, which will lead to differences in the time and temperature used by different manufacturers [15]. As a result, the nutritional composition of *D. officinale* is inevitably affected. Therefore, it is of great significance to explore the changes in *D. officinale* after processing.

However, at present, research on the processing of *D. officinale* mainly focuses on the drying methods [15] and always comprehensively evaluates the methods by the content of polysaccharide, flavonoid [16] and some other chemical composition indices of *D. officinale* combined with productivity efficiency. *D. officinale*, as a medicinal and edible plant, exerts pharmacological activities with multicomponent and multitarget properties. Thus, the total content of indices can only reflect the difference in individual components rather than the quality of the Chinese medicinal materials [17].

Metabolomics is a technology that can systematically identify and quantify metabolites [18], with the characteristics of high throughput and high sensitivity as proteomics and transcriptomics [19,20,21]. It can reveal the effect of external or internal disturbance on plant metabolism by characterizing the changes in metabolites during the plant’s growth process, which is consistent with the holistic view of traditional Chinese medicine [22]. However, due to the complex metabolic changes in plants, there are still many kinds of metabolites with significant differences that can be screened out, which will result in great difficulty in identifying the metabolites with pharmacodynamic activity [23]. As a consequence, it is difficult to accurately reflect the quality changes in medicinal materials. Network pharmacology is a method that can be used to investigate how drugs work through the construction of a “component–target–disease” network [24]. By analyzing the complex and multilevel interaction networks, network pharmacology has become a comprehensive and efficient tool to decipher the multitarget mechanism of traditional Chinese medicine, and it can be applied to search for compounds with pharmacodynamic activity. Molecular docking is a computer-aided drug design technique based on a computer-simulated structural approach that aids in the prediction of how compounds may interact with biological targets [25]. Through molecular docking, the binding mode and affinity of active components to important targets are verified to increase the accuracy of network pharmacological predictions [26].

**Figure 1:**
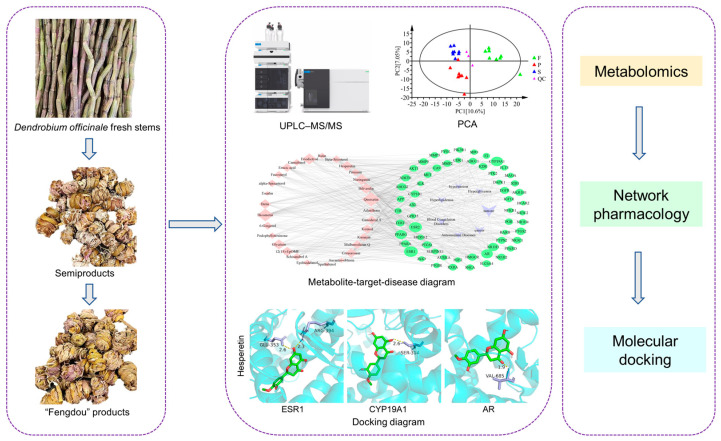
Experimental flow chart.

In this study, the strategy of targeted metabolomics combined with network pharmacology and molecular docking was adopted to explore the effect of processing on the quality of *D. officinale*. The metabolites of the materials were determined by ultra-performance liquid chromatography–mass spectrometry (UPLC-MS/MS) technology. Multivariate statistical analysis methods were employed to analyze the data, and metabolites with a significant difference were screened, which could reflect the changes in *D. officinale* metabolism after processing. Then, the network pharmacology method was used to analyze the differential metabolites, and the metabolite–target–disease network was constructed by Cytoscape. By analyzing this network, differential metabolites with pharmacodynamic activity could be screened. Finally, computer-aided validation of the network pharmacology results was performed using molecular docking techniques. The overall flow is shown in Figure 1.

## 2. Materials and Methods

### 2.1. Chemicals and Reagents

Methanol, acetonitrile and formic acid were UPLC-MS-grade reagents purchased from CNW Technologies (Düsseldorf, Germany). Phenol, sulfuric acid, ethanol, aluminum nitrate (Al(NO_3_)_3_), sodium nitrite (NaNO_2_) and sodium hydroxide (NaOH) were analytical-grade reagents purchased from Xilong Scientific Co., Ltd., (Guangdong, China).

### 2.2. Materials

#### 2.2.1. Raw Materials

The *D. officinale* stems were purchased from the Jinxingwang Officinale Planting Professional Cooperative (Zhejiang, China). The stems used in this study were from the same batch. The *D. officinale* samples were three years old and grown in Shuangfeng Township (bounded by 111°51′43″–112°31′7″ east longitude and 27°12′31″–27°41′51″ north latitude), which is located in Dajing, Leqing, Zhejiang Province, China. The processing of *D. officinale* is mainly based on the method listed in the 2015 edition of *Chinese Pharmacopoeia*: take the dried stems of *D. officinale*, remove impurities, cut off part of the fibrous roots, twist them into spiral or spring shape while heating and dry them. The detailed process is as follows. Stems were harvested using scissors, leaves were removed, stems were washed with tap water and stems were drained in a dry place and then tested as fresh samples (F). The *D. officinale* stems were placed into a bamboo oven, baked overnight (12 h) at 100 °C to make the fresh stems soft and cut into segments with lengths of 5 cm. The segments were rolled into spiral shapes, wrapped with kraft paper strips and baked at 100 °C in the oven for a short period (about 5 min) for the first finalization, obtaining the semiproduct samples (S). Baking was continued for 24 h until the *D. officinale* stems had dried out. The kraft paper was removed to obtain the “Fengdou” product (P). An HB43-S Moisture Analyzer (Mettler Toledo, Zurich, Switzerland) was used to determine the initial moisture content.

#### 2.2.2. Pretreatment

The samples were frozen at −20 °C for 4 h and transferred to −80 °C for 24 h. The freeze-dryer (SCIENTZ-10 N, Ningbo Scientz Biotechnology Co., LTD., Zhejiang, China) was used for drying the samples. The drying process was conducted under 1 Pa of absolute pressure until the moisture content reached 0.08 ± 0.03 g H_2_O/g d.w. The cold trap and heating plate were maintained at temperatures of −68.5 °C and 27 °C, respectively. Following drying, the samples were ground into powder using a muller and sifted through an 80-mesh sieve before being stored in a dry location for later analysis.

### 2.3. Determination of the Main Medicinal Components

#### 2.3.1. Total Polysaccharide Content of *D. officinale*

Ultrasonic-assisted extraction was used to process the samples, and the total polysaccharide content was assessed through the phenol-sulfuric acid method. Specifically, take *D. officinale* powder in a 100 mL centrifuge tube, add distilled water according to the material–liquid ratio of 1:120 (m/V), mix well and ultrasound (50° C, 240 W) for 2 h. Filter immediately after the ultrasound, take a small amount of distilled water to wash the filter residue in batches and combine the filtrate. Transfer the filtrate to a 100 mL volumetric flask for bandwidth evaluation. Precisely measure 5 mL in a 50 mL centrifuge tube, add 25 mL of absolute ethanol, shake well, refrigerate at 4 °C for 4 h, then centrifuge (10,000 r/min, 20 min), discard the supernatant, take 20 mL of 80% ethanol to wash the pellet, 8000 r/min centrifuge for 15 min and wash twice. Discard the supernatant, the resulting pellet is crude polysaccharide, evaporate the solvent and add water to dissolve and carry out a bandwidth evaluation (50 mL). Then, 4.0 mL of 240-fold diluted samples was mixed with 1.0 mL phenol solution (50 g L^−1^) and thoroughly blended. Subsequently, the mixture was reacted for 30 min at room temperature after adding 5.0 mL of concentrated sulfuric acid. The UV–visible spectrophotometer (UV-5500PC, Shanghai, China) was used to take the absorbance measurement of the reaction mixture at 490 nm. Each experiment was repeated in triplicate, and the outcomes were expressed as milligrams of D-glucose equivalent (GE) for each gram of sample.

#### 2.3.2. Total Flavonoid Content of *D. officinale*

The samples were processed by ultrasonic-assisted extraction, and the content of total flavonoid was detected by the Al(NO_3_)_3_-NaNO_2_-NaOH colorimetric method with slight modification. Specifically, take 0.50 g of *D. officinale* powder sample into a 50 mL centrifuge tube, add 25 mL of 80% ethanol aqueous solution and shake quickly to fully infiltrate the sample with the extract. Place it in an ultrasonic cleaner (55° C, 400 W) for 40 min to fully extract the sample. After finishing, centrifuge at 8000 r/min pieces for 8 min to retain the supernatant. A mixture of 0.2 mL NaNO_2_ solution (50 g L^−1^) and 1.0 mL of 50-fold diluted samples was shaken for 30 s and reacted for 6 min. Then, 0.2 mL Al(NO_3_)_3_ solution (100 g L^−1^) was added and reacted for 6 min before finally adding 0.8 mL NaOH solution (40 g L^−1^) and reacting for 10 min. The SP-Max 3500FL universal microplate reader (Shanghai) was used to measure the absorbance of the reaction mixture at 506 nm. Each experiment was repeated in triplicate and the outcome was expressed as milligrams of rutin equivalent (RE) for each gram of sample.

### 2.4. Targeted Metabolomics Based on UPLC-MS/MS

#### 2.4.1. Sample Preparation

Individual samples of 50 mg were weighed meticulously and moved to an Eppendorf tube. Methanol/water extract solution at a ratio of 3:1 (prechilled to −40 °C, containing internal standard 2-chloro-l-phenylalanine) was added in an amount of 700 μL. The samples were vortexed for 30 s, then homogenized at 40 Hz for 4 min, followed by 5 min of sonication in an ice-water bath. This procedure was conducted thrice. The samples were then subjected to extraction overnight at 4 °C on a shaker. The resulting samples were centrifuged for 15 min (12,000 rpm, 4 °C). The supernatant was meticulously permeated through a microporous 0.22 μm membrane and diluted tenfold with a methanol/water mixture (*v*:*v* = 3:1). The solution was vortexed for 30 s and transferred to 2 mL glass vials. As a quality control (QC) sample, 20 μL was taken from each sample and collated. The samples were stored at −80 °C until UPLC-MS/MS analysis.

#### 2.4.2. UPLC-MS/MS Analysis

An EXIONLC System from Sciex was utilized for UPLC separation. Mobile phase A constituted of 0.1% formic acid in water, while mobile phase B was acetonitrile. Gradient elution conditions are shown in Table 1.

The temperature of the column was set at 40 °C. The autosampler temperature was established at 4 °C and the injection volume was set at 2 μL. To develop the assay, a Sciex QTrap 6500 + from Sciex Technologies was employed. The ion source parameters were as follows: ion spray voltage: +5500/−4500 V, curtain gas: 35 psi, temperature: 400 °C, ion source gas 1: 60 psi, ion source gas 2: 60 psi and DP: ± 100 V.

#### 2.4.3. Data Preprocessing and Annotation

Data acquisition and processing for Multiple-reaction-monitoring (MRM) was carried out using SCIEX Analyst Work Station Software (Version 1.6.3). MS-converter was used to change MS raw data (.wiff) files to the TXT format. An in-house R program and database were applied for peak detection and annotation. The data were standardized using the internal standard normalization method.

#### 2.4.4. Statistical Analysis

The data were analyzed using GraphPad Prism 8 (GraphPad Software, San Diego, CA, USA). Analysis of variance (ANOVA) and Duncan multiple comparison tests were performed using SPSS software (version 26.0, IBM, Chicago, IL, USA). Principal component analysis (PCA) and orthogonal partial least squares discriminant analysis (OPLS-DA) of metabolomics data were performed using SIMCA-P software (Version14.1, Umetrics, Umea, Sweden), and 200 permutation tests were conducted on the OPLS-DA model to determine whether the model was overfitting. Metabolites with significant differences were screened according to the fold change (FC) value and the variable important in projection (VIP).

### 2.5. Network Pharmacology Analysis

#### 2.5.1. Target Genes of Metabolites

The TCMSP database, whose full name is Traditional Chinese Medicine Systemic Pharmacology Database and Analytical Platform, is a classical herbal systemic pharmacology platform that captures the relationship between chemicals, targets and diseases [27]. The TCMSP database was used to search for the oral bioavailability (OB) [28] and drug-likeness (DL) [29] of the significantly differential metabolites. The threshold was set as OB ≥ 20% and DL ≥ 0.10 to screen out metabolites with potential medicinal properties. Then, these metabolites were searched by the PubChem database, reserving the information of SMILES and InChI in an Excel file. Afterward, the SMILES were input into the Swiss Target Prediction database [30,31] and SEA database [32], and the InChI files were input into the BATMAN database [33] to predict the corresponding targets of the metabolites. Preserving the targets with the species was set as “homo sapiens” and possibility > 0 in the Swiss Target Prediction database [34]; the species information was human, *p* < 0.05 in the SEA database and score > 15 in the BATMAN database. Duplicate values were merged and removed.

#### 2.5.2. Target Genes of Diseases

Based on the fact that *D. officinale* has physiological activities such as anticancer, hypoglycemic, antihyperlipidemic, antihypertension, anticoagulant, tumor-inhibiting and immunity-improving activities, according to the references, we searched the target files of these diseases using the DisGeNET database [35], GeneCards database [36] and OMIM database. The targets with score ≥ 0.1 in the DisGeNET database, the top 300 targets in GeneCards and the targets with * in the OMIM database were selected for further analysis. Duplicate values were merged and removed.

#### 2.5.3. Network Construction

A Venn diagram of the targets of metabolites and diseases was constructed to obtain the common targets [37]. Then, the metabolite–target–disease network was constructed by Cytoscape software (Version 3.8.2). By analyzing this network, the parameters of degree, betweenness and closeness were obtained [38]. Next, the average of these three parameters was set as the threshold to screen out the metabolites with large effects and influences.

### 2.6. Molecular Docking

The 3D structures of metabolites were downloaded in SDF format from PubChem (https://pubchem.ncbi.nlm.nih.gov/ (accessed on 8 November 2022)) and imported into Open Babel software (Version 3.1.1) to convert them into mol2 format files. Target protein structures were downloaded in PDB format from RCSB PDB (http://www.rcsb.org/ (accessed on 8 November 2022)) and imported into PyMOL (version 2.6.0). Then, water molecules, ions and small molecule ligands were removed and saved in PDB format. Hydrogen was added to the target protein using AutoDock Tools (version 1.5.7), and the target protein and metabolites were converted to PDBQT format for molecular docking using AutoDock Vina (version 1.1.2) [39]. In this study, the method of blind docking was used for molecular docking, and the grid box size was set as the whole model. Other main operating parameters include exhaustiveness = 12, energy range = 4, num modes = 20. The conformation with the best affinity was used as a criterion to screen possible molecular docking conformations. PyMOL was used to visualize the outcomes. Finally, the original ligand was separated from the protein and redocked for verification [40]. If the root mean square deviation (RMSD) between the docked ligand small molecule and the original ligand small molecule is less than 2 Å, it indicates that the docking method can well reproduce the original binding mode of the ligand and receptor. At the same time, the docking method and parameter selection are reasonable, and the docking result is highly reliable.

## 3. Results and Discussion

### 3.1. Effects of Processing on the Contents of Total Polysaccharide and Flavonoid in D. officinale

**Figure 2:**
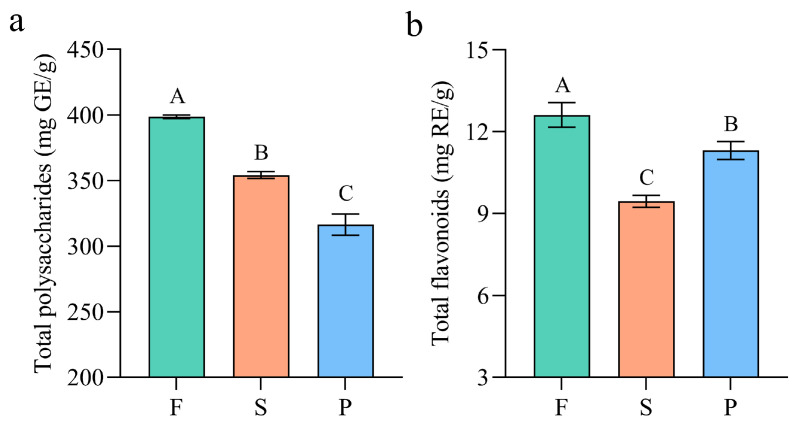
The total polysaccharide (**a**) and total flavonoid (**b**) contents of *D. officinale* stem at different processing stages. F: fresh stems; S: semiproducts; P: “Fengdou” products. GE and RE represent glucose equivalent and rutin equivalent, respectively. Error bars represent standard deviation. The different capital letters (A, B and C) indicate significant differences among groups (*p* < 0.01).

Polysaccharides and flavonoids are important medicinal components of *D. officinale*. *D. officinale* polysaccharide is a heteropolysaccharide containing different components, mainly composed of mannose, glucose, galactose, xylose, arabinose, rhamnose, fructose and galacturonic acid [41,42]. The polysaccharides in *D. officinale* that have been reported so far include DOP-1-A1, DOP, HDOP, DOPW-1, LDOP, S32 and so on [41,43]. *D. officinale* polysaccharide has multiple pharmacological activities such as antioxidant, anti-inflammatory and anticancer [43]. The flavonoids in *D. officinale* have been reported to consist of three main groups, namely flavonoids, flavonols and dihydro-flavonoids [44]. They have powerful antioxidant and free-radical scavenging abilities [45]. The contents of total polysaccharide and flavonoid in the F, S and P samples were determined, and the results were analyzed by Duncan’s multiple comparative analysis, as shown in Figure 2. The contents of polysaccharide in F, S and P were 398.60 mg/g, 354.20 mg/g and 316.47 mg/g, respectively, as illustrated in Figure 2a, indicating a decreasing trend with a significant difference (*p* < 0.01). This might be caused by the degradation of macromolecular polysaccharides under high temperature during processing. Similarly, as depicted in Figure 2b, the flavonoid content decreased significantly during the processing of F into S (*p* < 0.01), which could likely be attributed to the rapid degradation of thermolabile flavonoids at high temperature. The flavonoid content showed a slight increase from S to P, which is consistent with the result of Ravisankar, due to the destruction of tissue of *D. officinale* [46]. The results preliminarily indicated that processing could lead to significant changes in the polysaccharide and flavonoid contents of *D. officinale*.

### 3.2. Targeted Metabolomics Analysis of D. officinale Stems at Different Processing Stages

UPLC-MS/MS technology was employed to determine the metabolic components in the stems of *D. officinale*, and 970 signal peaks were found, from which 628 metabolites were identified. The metabolites were classified into 10 classes, with flavonoids, terpenes, alkaloids, phenylpropanoids, phenolic acids, and their derivatives accounting for 83.2% of the total metabolites.

#### 3.2.1. Multivariate Statistical Analysis

**Figure 3:**
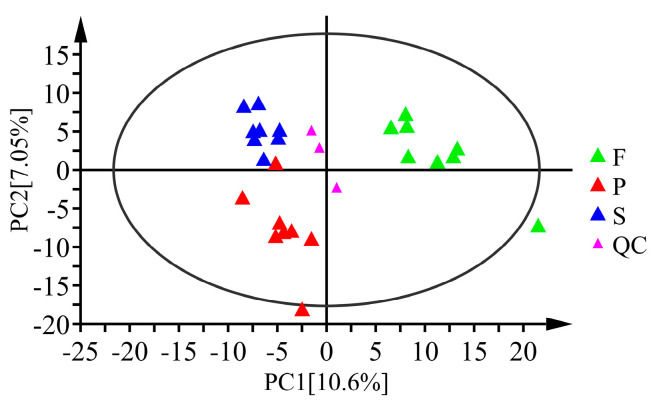
Score plots of PCA for *D. officinale* samples of F, S, P and quality control (QC) samples. PC1 and PC2 represent the first and second principal components, respectively.

PCA was performed on the samples to reflect the status of the original data (Figure 3). Each group of samples had a good clustering effect within the group, and there was an apparent tendency of separation between groups. These results indicated that the repeatability of samples was good, and there were significant differences among samples in different groups, demonstrating that processing had a considerable impact on the metabolism of *D. officinale*. Moreover, the groups F and P had a clear separation trend in the first principal component, while the groups S and P separated evidently. This illustrates that the effect of the first stage of processing on the metabolism of *D. officinale* was more intense than that of the second stage.

**Figure 4:**
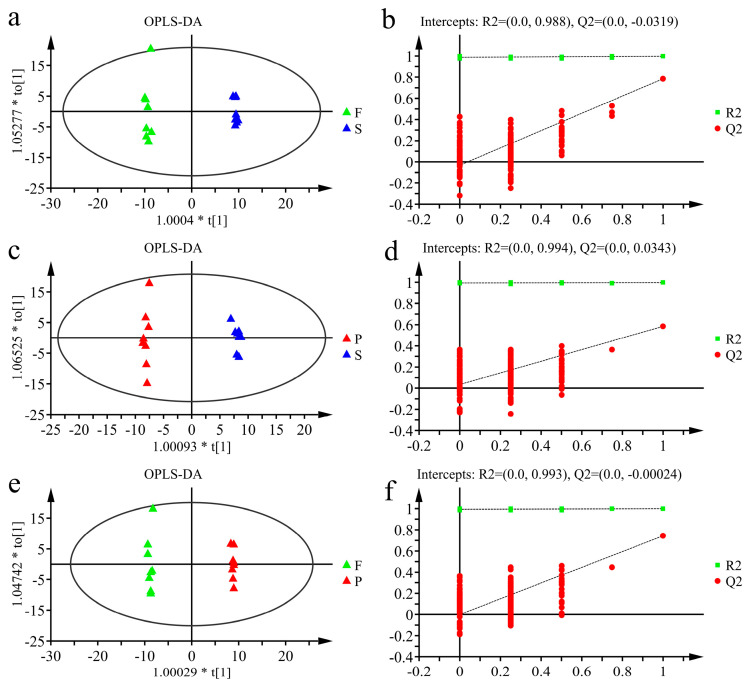
(**a**,**c**,**e**) are the score plots of the OPLS-DA model; (**a**): F vs. S; (**c**): P vs. S; (**e**): F vs. P. The *X*-axis and *Y*-axis represent the predictive principal component and the orthogonal principal component, respectively. The plots of (**b**,**d**,**f**) are the model validation of (**a**,**c**,**e**), respectively.

To minimize the interference of irrelevant variables and find the differences between groups, OPLS-DA was employed to analyze the data. OPLS-DA models of F vs. S, S vs. P and F vs. P were established (Figure 4a,c,e), and the R^2^ and Q^2^ values of the models were 0.998 and 0.785, 0.997 and 0.584, and 0.999 and 0.743, respectively, indicating that the established model could explain the data matrix information and had good predictive ability. The OPLS-DA model was verified by a sevenfold cross-validation method and 200 permutation tests. The results shown in Figure 4b,d,f illustrated that the OPLS-DA models were meaningful and that the VIP values obtained based on the model were reliable and could be used for further analysis.

#### 3.2.2. Identification of Differential Metabolites

The metabolites with significant differences among groups were screened using VIP parameters of the OPLS-DA model combined with FC values of univariate statistical analysis (VIP ≥ 1, |log_2_ (FC)| ≥ 1). A total of 109 differential metabolites were screened. The classification results are shown in Table 2. There were 79, 34 and 51 differential metabolites in the comparisons of F vs. S, S vs. P and F vs. P, respectively. Obviously, the number of metabolites with significant differences in the comparison of F vs. S was the highest, while the number of metabolites screened between S and P was the lowest. Compared with the second stage of processing (from S to P), the metabolic fluctuation of *D. officinale* was more dramatic in the first stage of processing (from F to S).

Table 2 shows that 62 metabolites were significantly decreased in the S group compared with the F group, while 17 metabolites were upregulated. The metabolites whose content decreased mainly included flavonoids (12), terpenoids (13), steroids and their derivatives (7), alkaloids (6) and phenylpropanoids (6). The flavonoids whose content was significantly downregulated were mainly oxycarbidosine flavonoids, such as rutin and kaempferol glycoside, which might be related to the destruction of the structure of flavonoids under prolonged thermal processing [16]. Most of the terpenoids that were significantly downregulated were triterpenoids, which might be due to their tendency to break bonds during thermal processing, leading to the degradation of metabolites.

In contrast to the results of the first processing stage, 32 out of 34 metabolites were upregulated in the P group compared with the S group. This could be attributed to the degradation of macromolecules in *D. officinale* and the production of metabolites with better thermal stability during the second processing stage. Additionally, it might be related to the rupture of cells under high temperature.

### 3.3. Network Pharmacology Analysis

**Figure 5:**
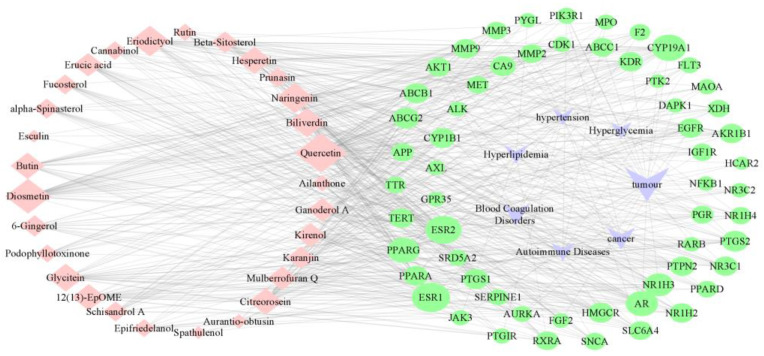
Metabolite–target–disease diagram. Pink nodes represent metabolites, green nodes are targets and purple nodes are diseases. Edges in the network are used to connect metabolites and targets, and diseases and targets. The size of nodes depends on their degree value; the larger the node is, the greater the degree value.

To explore the effect of processing on the pharmacological activity of *D. officinale*, the differential metabolites were analyzed by network pharmacology. According to the parameters of OB and DL (Table S1), a total of 29 metabolites with potential drug properties were screened, and 1678 gene targets were matched by the public database. In addition, 1892 gene targets were matched for the seven diseases. Taking the intersection of metabolite-related targets and disease-related targets, a total of 59 common targets were obtained. Then, the compound–target–disease network shown in Figure 5 was constructed. This network contained 95 nodes and 700 edges, indicating that the physiological activity of *D. officinale* stems was the result of the joint action of multiple components and multiple targets. Using Cytoscape to analyze this network, the parameters of degree, betweenness and closeness of the nodes were obtained. The degree value of a node was reflected in the network’s node size. The size of the node increased with the degree value, indicating the node’s importance in the network.

The average of the three parameters was used as the threshold to screen the core nodes. As a result, 14 kinds of key nodes were screened, including quercetin, diosmetin, eriodictyol, naringenin, biliverdin, glycitein, hesperetin, 12(13)-EpOME, butin, citreorosein, kirenol, ganoderol A, mulberrofuran Q and erucic acid. The 14 metabolites might be the material basis for the physiological activity of *D. officinale*, and their contents fluctuated significantly during processing. Processing inevitably affects the physiological activity of *D. officinale*. Nine gene targets, including ESR1, ESR2, CYP19A1, AR, NR1H3, PTGS2, PPARG, PTGS1 and CA9, were selected because they played an important role in the network. In addition, the node representing the tumor was obtained, which indicated that the metabolic fluctuation caused by the processing of *D. officinale* might have a great influence on the physiological activity in tumor inhibition.

### 3.4. Molecular Docking Analysis

Among the nine important gene targets screened by the network pharmacology compound–target–disease network, there were five important targets related to three diseases of hyperglycemia, coagulation disorders and tumors, including ESR1, CYP19A1, AR, PTGS2 and PPARG. Seven key metabolites, eriodictyol, hesperetin, naringenin, butin, diosmetin, citreorosein and mulberrofuran Q, were selected for molecular docking analysis with ESR1, CYP19A1, AR, PTGS2 and PPARG, respectively.

**Figure 6:**
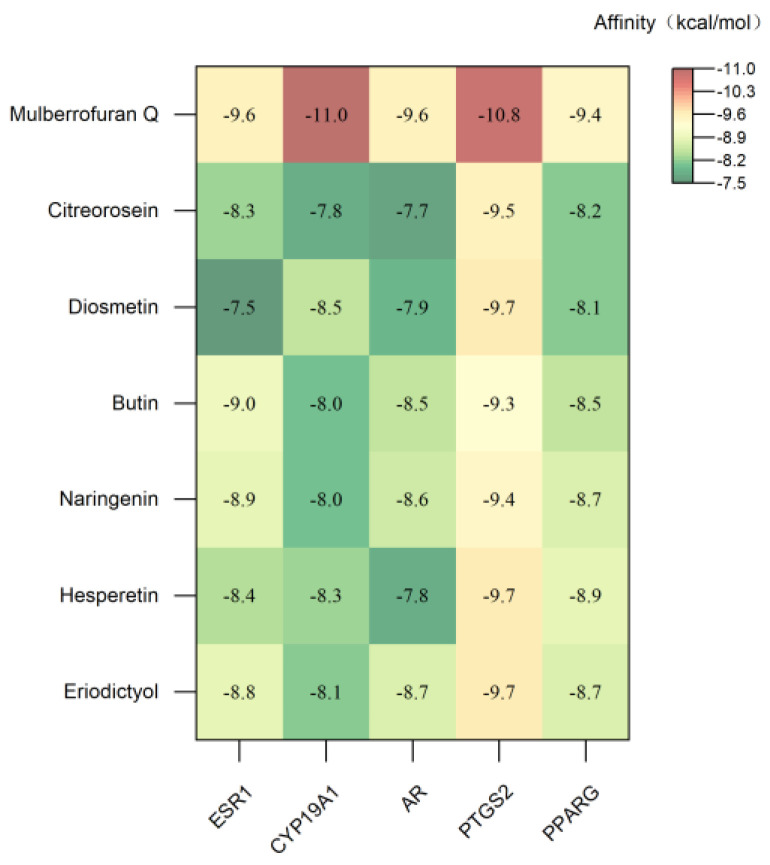
Heatmap of molecular docking affinity.

**Figure 7:**
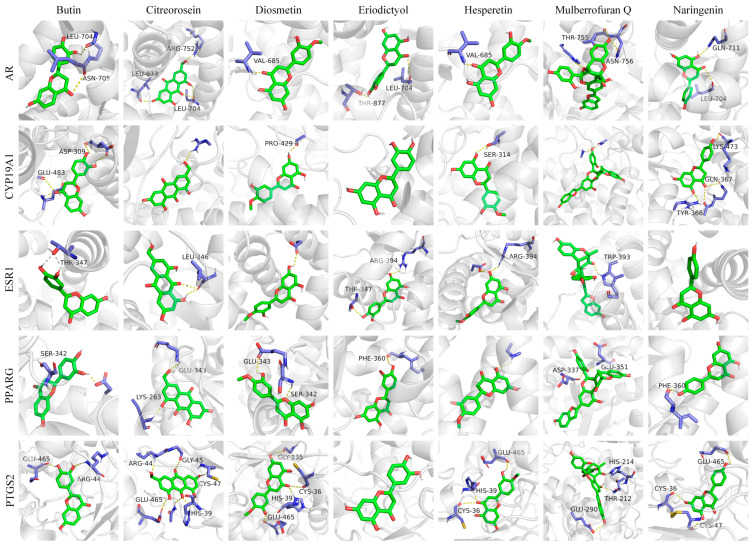
Docking diagram of key metabolites and core targets.

A negative affinity value indicates that the ligand and the receptor can bind spontaneously, and the smaller the binding energy, the higher the affinity activity of the ligand and the receptor, and the better the molecular docking binding effect [47]. According to the conventional view, binding affinities less than −5 and −7 kcal mol^−1^ indicate good and strong binding activity, respectively [48]. The molecular docking results are shown in Figure 6 and Figure 7.

The binding energies of the seven key metabolites to ESR1, CYP19A1, AR, PTGS2 and PPARG were all below −7 kcal mol^−1^, indicating that the seven key metabolites could spontaneously bind to the targets to form target–ligand binary complexes with good binding activity. Additionally, hydrogen bonding played a major role in the binding of these metabolites and amino acid residues. As shown in Table S2, the RMSDs of the original ligands of the five key target proteins after docking to the proteins were less than 2 Å, indicating that the docking methods and parameters were reasonably designed and that the docking results were highly reliable.

### 3.5. Metabolite Relative Content Analysis

**Figure 8:**
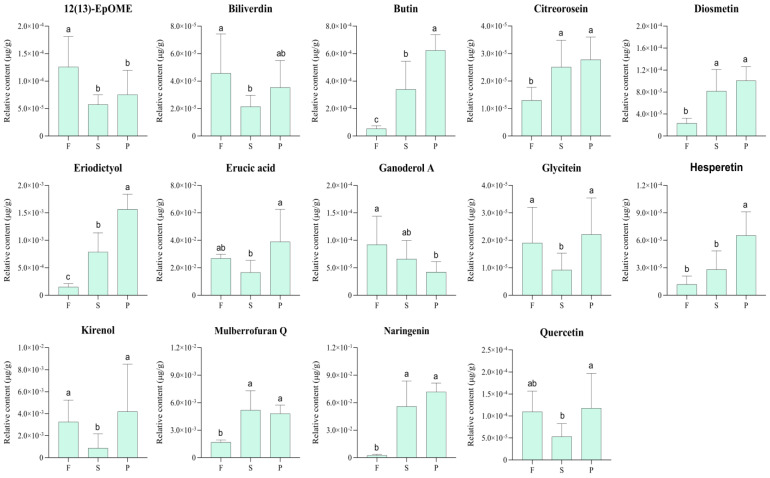
The relative abundance of 14 differential metabolites with potential pharmacological activities at different processing stages of *D. officinale*. Error bars represent standard deviation. The different capital letters (a, ab, b and c) indicate significant differences among groups (*p* < 0.05).

The relative amounts of the 14 selected key metabolites with potential physiological activities of *D. officinale* in different processing stages were compared, as shown in Figure 8.

The contents of eriodictyol, hesperetin, naringenin, citreorosein, bilirubin and diosmetin showed a continuously increasing trend during the processing of *D. officinale*. The content of mulberrofuran Q increased in the first processing stage and then decreased slightly in the second processing stage. Overall, the content of the mulberrofuran Q in the P samples was higher than those in the F samples. The contents of glycitein, quercetin, kirenol, 12(13)-EpOME, erucic acid and biliverdin decreased at first and then increased. The content of the first three metabolites was basically equal in the F and P groups, and erucic acid decreased and then increased to a level higher than that of fresh samples. The contents of 12(13)-EpOME and biliverdin decreased after the first stage of processing and then increased slightly in the second stage but were still lower than those of fresh samples. Among the 14 metabolites, only ganoderol A decreased continuously throughout the process. In conclusion, a total of seven metabolites, including eriodictyol, hesperetin, naringenin, butin, diosmetin, citreorosein and mulberrofuran Q, were selected, and their contents were clearly increased in the P group compared with those in the F group, while the contents of 12(13)-EpOME, biliverdin and ganoderol A were decreased significantly in the P group compared with those in the F group.

To explore the effect of processing on the pharmacological activity of *D. officinale*, the targets and diseases associated with 14 key metabolites in the PPI network were sorted out based on the results of network pharmacological analysis in Section 3.3, and the results are shown in Table S3. There were 28, 23, 27, 22, 34, 22 and 18 targets associated with eriodictyol, hesperetin, naringenin, butin, diosmetin, citreorosein and mulberrofuran Q, respectively. These seven metabolites might have potential anticoagulant, hypoglycemic and tumor inhibition physiological activities by acting on these targets. Molecular docking analysis initially validated the idea that these seven metabolites had good affinity for five important gene targets, including ESR1, CYP19A1, AR, PTGS2 and PPARG, which are associated with hyperglycemia, blood-clotting disorders and tumors.

The finding that naringenin is the most important active ingredient of *D. officinale* is consistent with previous studies, which have shown that naringenin has strong binding activity with the core targets PTGS2 and PPARG and may exert anti-tumor and therapeutic hyperlipidemia effects by modulating the PTGS2 and PPARG targets [49,50], which is similar to the results of the present experiment. In addition, studies have shown that naringenin is a modulator of AR and PPARG targets [51,52]. Moreover, diosmetin is a potent inhibitor of PPARG [51] and CYP1A1 [53] targets and an agonist of ESR [54]. Hesperetin has antioxidant, anti-inflammatory and antibacterial activities [55]. Using bioinformatics and in vitro research methods, Adam et al. found that hesperetin modulates PPARG expression and has potential for the treatment of breast cancer [56]. Citreorosein is a natural anthraquinone derivative that reduces PTGS2 expression [57]. Butin has strong antioxidant and anti-inflammatory effects [58]. It has been reported that butin can inhibit CYP19 activity [59]. Eriodictyol is an alpha-glucosidase inhibitor [60] with hypoglycemic effects that significantly control obesity and diabetes [61]. It has been reported that eriodictyol stimulates adipogenesis, increases PPARG mRNA expression and increases PPARG protein levels [62]. However, it is noteworthy that little has been reported about the activity of mulberrofuran Q in *D. officinale*. Therefore, mulberrofuran Q deserves attention and more in-depth research in the future. The above results are similar to the results of molecular docking experiments, indicating that the seven key metabolites may exert potential pharmacological effects through five important targets including ESR1.

Combined with the variation in content, it could be concluded that processing could enhance the anticoagulant, hypoglycemic and tumor inhibition activities of *D. officinale*. In addition, there were 22, 26 and 18 targets associated with 12(13)-EpOME, biliverdin and ganoderol A, respectively, which are also associated with anticancer, lipid-lowering and tumor inhibition activities. It seemed to indicate that the processing of *D. officinale* could lead to a reduction in anticancer, antihyperlipidemia and tumor inhibition activities. However, Figure 8 shows that the relative amounts of 12(13)-EpOME and ganoderol A were only 1.0 × 10^−4^ μg/g and that of biliverdin was only 4.0 × 10^−5^ μg/g, which were very low and had little or even negligible influence on the efficacy and activity of *D. officinale*. In general, the content of active metabolites in fresh *D. officinale* samples increased significantly after processing. As a result, the anticoagulant, hypoglycemic and tumor inhibition activities could also be improved, theoretically.

## 4. Conclusions

In this study, targeted metabolomics combined with network pharmacology and molecular docking was employed to investigate the change in metabolism and the effect of processing on the quality of *D. officinale*. The content of polysaccharide decreased significantly during processing, while the content of flavonoid decreased first and then increased, indicating that processing inevitably affected the quality of *D. officinale*. Then, targeted metabolomics was used to characterize the metabolites. A total of 628 metabolites were detected, and 109 differential metabolites were screened by univariate analysis combined with multivariate analysis. A total of 79, 34 and 51 differential metabolites were screened from the F-S, S-P and F-P comparison groups, respectively. Then, the network pharmacology method was used to analyze the differential metabolites, and 29 differential metabolites were screened out as they have potential pharmacological activity. Combined with seven diseases, 59 targets were identified as the key target of compounds. The metabolite–target–disease network was constructed by Cytoscape, and 14 compounds were screened out. The results of changes in the relative abundance of metabolites showed that there were seven metabolites, including eriodictyol, hesperetin, naringenin, bilirubin, diosmetin, citreorosein and mulberrofuran Q, which have potential anticoagulant, hypoglycemic and tumor inhibition activities, with an increasing trend in the “Fengdou” product. The results suggest that processing can increase the content of some active metabolites of *D. officinale* and improve its efficacy. This study provides new insight into the effect of processing on the medicinal quality of *D. officinale* and provides a solid theoretical basis for the processing and application of *D. officinale* in pharmaceuticals.

## Figures and Tables

**Table 1:** Parameter of gradient elution.

Time/min	Mobile Phase A/%	Mobile Phase B/%
0	98	2
0.5	98	2
10	50	50
11	5	95
13	5	95
13.1	98	98
15	98	98

**Table 2:** The classification of 109 differential metabolites.

Class	F vs. S		S vs. P		F vs. P	
	Down	Up	Down	Up	Down	Up
Flavonoids	12	8	0	6	0	9
Terpenoids	13	2	0	5	3	3
Alkaloids	6	4	0	7	5	9
Coumarins	6	0	1	2	0	2
Phenols	7	1	0	3	4	3
Lipids	5	1	0	2	4	1
Steroids	8	0	0	4	2	0
Anthraquinones	0	0	1	0	1	1
Organic acids	3	0	0	0	2	0
Others	2	1	0	3	0	2
Significantly differential metabolites	62	17	2	32	21	30
Total	109					

Note: up/down represents that the metabolite content of the latter group has a changing trend compared with the previous group. F: fresh stems; S: semiproducts; P: “Fengdou” products.

## Data Availability

The data presented in this study are available on request from the corresponding author. The data are not publicly available due to restrictions.

## Supplementary Materials

The following supporting information can be downloaded at: https://www.mdpi.com/article/10.3390/metabo13080886/s1, Table S1: Information on differential metabolites of *D. officinale* that have potential physiological activity; Table S2: Molecular docking information of target proteins and original ligands; Table S3: Information on differential metabolites of *D. officinale* that have potential physiological activity.
