# Supplementary material for: Integrated Analysis of Metabolomics Combined with Network Pharmacology and Molecular Docking Reveals the Effects of Processing on Metabolites of Dendrobium officinale
Source: Metabolites. 2023 Jul 26;13(8):886. doi: 10.3390/metabo13080886 (PMC10456568; doi:10.3390/metabo13080886)
Supplement: Supplementary file 1 [file metabolites-13-00886-s001.zip › metabolites-2486131-supplementary.pdf]

Table S1. Information on differential metabolites of *D. officinale* that have potential physiological activity.

Name	OB（%）	DL	Name	OB（%）	DL
Quercetin	46.43	0.28	Diosmetin	31.14	0.27
Biliverdin	23.79	0.75	6-Gingerol	35.64	0.16
Naringenin	59.29	0.21	Podophyllotoxinone	49.61	0.86
Prunasin	26.9	0.18	Glycitein	50.48	0.24
Hesperetin	70.31	0.27	12(13)-EpOME	37.63	0.19
Beta-Sitosterol	36.91	0.75	Schisandrol A	24.16	0.58
Rutin	69.94	0.21	Epifriedelanol	25.19	0.76
Eriodictyol	71.79	0.24	Spathulenol	80.01	0.12
Cannabinol	22.04	0.32	Aurantio-obtusin	31.55	0.37
Erucic acid	28.56	0.26	Citreorosein	22.19	0.27
Ailanthone	27.96	0.74	Mulberrofuran Q	69.49	0.14
Fucosterol	43.78	0.76	Karanjin	69.56	0.34
alpha-Spinasterol	42.98	0.76	Kirenol	28.38	0.35
Esculin	20.43	0.36	Ganoderol A	44.69	0.8
Butin	69.94	0.21			

Table S2. Molecular docking information of target proteins and original ligands.

Target protein	PDB ID	Original ligand	RMSD/Å
ESR1	6IAR	H8W	0.001
CYP19A1	3S79	ASD	0.000
AR	5VO4	9FG	0.246
PTGS2	5F1A	COH	0.000
PPARG	6D8X	EDK	0.879

Table S3. Information on differential metabolites of *D. officinale* that have potential physiological activity.

No.	Name	The number of associated targets	Potential physiological activity
1	Quercetin	40	anticancer, hypoglycemic, antihyperlipidemia, antihypertension, anticoagulant, tumor inhibition and improving the immune activity
2	Diosmetin	34	hypoglycemic, anticoagulant, tumor inhibition
3	Eriodictyol	28	hypoglycemic, antihyperlipidemia, anticoagulant and tumor inhibition
4	Naringenin	27	hypoglycemic, anticoagulant, tumor inhibition
5	Biliverdin	26	hypoglycemic, anticancer, antihyperlipidemia, tumor inhibition, antihypertension and anticoagulant
6	Glycitein	24	anticancer, antihyperlipidemia, tumor inhibition
7	Hesperetin	23	hypoglycemic, anticoagulant
8	Butin	22	antihyperlipidemia, tumor inhibition, anticoagulant
9	12(13)-EpOME	22	anticancer, hypoglycemic, antihyperlipidemia, tumor inhibition and improving the immune activity
10	Citreorosein	22	hypoglycemic, anticoagulant, tumor inhibition
11	Kirenol	19	hypoglycemic, antihypertension and improving the immune activity
12	Erucic acid	18	antihypertension
13	Mulberrofuran Q	18	hypoglycemic, anticoagulant
14	Ganoderol A	18	tumor inhibition
